# Suckling a protein-restricted rat dam leads to diminished albuminuria in her male offspring in adult life: a longitudinal study

**DOI:** 10.1186/1471-2369-7-14

**Published:** 2006-09-29

**Authors:** Clive J Petry, Bridget J Jennings, Lynwen A James, Charles N Hales, Susan E Ozanne

**Affiliations:** 1 Department of Clinical Biochemistry, University of Cambridge, Addenbrooke's Hospital, Cambridge, UK; 2 Department of Paediatrics, University of Cambridge, Addenbrooke's Hospital, Cambridge, UK

## Abstract

**Background:**

Previous studies have shown that in male rats, exposure to *maternal *protein restriction either *in utero *or whilst suckling can have profound effects on both longevity and kidney telomere lengths. This study monitored albuminuria longitudinally in male rats whose mothers had been protein restricted either during pregnancy or lactation.

**Methods:**

Pregnant Wistar rats were fed either a 20% ('control') or an 8% protein ('low protein') diet. At two days of age some of the pups were cross-fostered to dams fed the diet that was *not *given to their biological mothers. At weaning all pups were fed standard chow. Urine samples were collected for the measurement of albumin and creatinine at monthly intervals from two months-of-age. Longitudinal analysis was then performed using repeated measures analysis of variance.

**Results:**

Overall estimated marginal geometric mean (95 % confidence interval) urine albumin to creatinine ratios were: control animals 79.5 (57.2~110.6) g/mol (n = 6 litters, 24 animals in total), those exposed *in utero *to maternal protein restriction 71.0 (47.4~106.5) (n = 4 litters, 16 animals in total), those exposed to maternal protein restriction whilst suckling 21.2 (14.7~30.4) (n = 5 litters, 20 animals in total) (p < 0.001). These latter animals had lower albumin to creatinine ratios than either of the two other groups (both p < 0.001), which had ratios that were indistinguishable from each other (p = 1.0). Similar results were gained using 24 h. urine albumin excretion rates. These differences became evident from three months-of-age and were long-lasting.

**Conclusion:**

Animals exposed to maternal protein restriction whilst suckling exhibited lower urine albumin excretions during much of adult life. As urine albumin can be nephrotoxic, these rats therefore appeared to be relatively protected against future nephron damage like that previously observed in animals exposed to maternal protein restriction *in utero*.

## Background

We have previously shown that maternal protein restriction in rats and mice at critical stages of development modulates lifespan of the offspring [1-4]. Using cross-fostering techniques, it was found that exposure to pre-natal maternal protein restriction followed by nursing by a control dam led to rapid 'catch-up' growth post-natally [5] and a reduced natural lifespan in male offspring in comparison to controls that were adequately nourished throughout development. Conversely exposure to maternal protein restriction whilst suckling was shown to lead to a slowing of growth [5] and an increased lifespan [1-4]. While the effects appeared to be stronger in males, possibly due to gender differences in renal antioxidant capacities [6], such trends were also evident in female offspring.

It was found that these alterations in longevity were associated with differences in age-related shortening of renal telomeres (the complexes of DNA and protein found at the end of chromosomes) [2]. Animals that were exposed to maternal protein restriction whilst suckling were shown to have slower rates of telomere shortening. Such changes in telomere length were not found in the liver and brain from these animals, implicating the kidney as being particularly vulnerable to the effects of the nutritional deficit at different stages of development. Since renal disease is a common cause of death in male rats [7], the finding of changes in kidney telomere length implicates altered renal function as a possible cause of the observed alterations in longevity. The present study was therefore designed to investigate whether there were differences in renal function in terms of albuminuria in male rats which were exposed to maternal protein restriction either *in utero *or whilst suckling.

## Methods

### Animals

All procedures involving animals were conducted under the Animals (Scientific Procedures) Act (1986). Virgin female Wistar rats with initial weights of 235–250 g, were maintained individually at 22°C on a controlled twelve hour light-dark cycle in standard cages in a conventional, non-barriered animal facility. They were mated and assumed to be pregnant when a vaginal plug was observed. From this time onwards they were randomly assigned one of two diets (both from Hope Farms, Woerden, The Netherlands). The control diet contained 20% (w/w) protein, 63.2% (w/w) carbohydrate, 4.3% (w/w) fat, 5% (w/w) vitamin and mineral mixture and 15370 kJ/kg energy, as described previously [5]. The low protein (isocaloric) diet contained 8% (w/w) protein, 76.2% (w/w) carbohydrate, 4.3% (w/w) fat, 5% (w/w) vitamin and mineral mixture and 15280 kJ/kg energy. Each dam remained on its respective diet throughout pregnancy and lactation. All animals were allowed to eat *ad libitum *throughout and had free access to water.

Offspring from a total of 11 mothers on the control diet and 5 mothers on the low protein diet were studied. Spontaneous delivery took place on day 22 of pregnancy. Mean litter sizes were 13 for rat dams fed either the 20% or isocaloric 8% protein diets. Control animals (n = 6 litters, 4 animals from each of them making a total of 24) were male offspring of dams fed the 20% protein diet throughout pregnancy and lactation. Two days after birth their litter sizes were standardised to eight pups (four males and four females, chosen randomly). Recuperated animals (n = 5 litters, 4 animals from each of 3 different litters and a further 2 from each of 2 different litters (making a total of 16), the lower number from these litters being due to one maternal low-protein-fed litter having a deficit of male offspring and one mother displaying standard infanticide behaviour [8]) were male offspring of dams who were fed the 8% protein diet. Two days after birth their litter sizes were standardised to four randomly chosen male pups and from this time until weaning they were cross-fostered and suckled dams fed the 20% protein diet. Post-natal low protein animals (PLP) (n = 5 litters, 4 animals chosen randomly from each of them making a total of 20) were male offspring of dams who were fed the 20% protein diet. Their litter sizes were not standardised (all male and female pups being allowed to reach weaning) but from two days after birth they were cross-fostered and suckled dams fed the 8% protein diet. All offspring (control, recuperated and PLP) were weaned onto a diet containing 19.6% (w/w) protein, 49.1% (w/w) digestible carbohydrate, 3.0% (w/w) fat, 3.4% (w/w) vitamin and mineral mixture and 14000 kJ/kg energy, at 21 days of age (LAD1 from Special Diet Services, Witham, U.K.). The study only investigated the male rats since these were the ones whose alterations in lifespan had previously reached statistical significance [1]. Each of these rats was weighed weekly.

### Laboratory analyses

Urine samples were collected from the rats housed in metabolic cages over 24 h. periods on a monthly basis. Samples were frozen at -20°C prior to analyses. Urine albumin concentrations were measured by enzyme linked immunosorbent assays [9] according to the manufacturer's instructions (using Rat Albumin kits from Immunodiagnostic Systems Ltd., Boldon, Tyne & Wear, U.K.). Creatinine concentrations were measured using a modified Jaffé reaction [10] (with kits supplied by Sigma Diagnostics, Poole, Dorset, U.K.). Monthly urine collections continued until the first rat had died (at ten months of age from natural causes, i.e. there was no major pathological cause of death being found at post-mortem examination). At this age blood samples (1 ml) were taken from the remaining animals' tails at the end of the last period of urine collection. The blood was centrifuged at 3,000 g for 10 min. and the plasma separated and frozen at -20°C prior to analyses. Plasma creatinine concentrations were measured to allow the calculation of creatinine clearances by multiplying the 24 h. urine volume by its creatinine concentration and dividing it by the plasma creatinine concentration. At this stage the animals were then killed in order to remove and then weigh their kidneys (full urine albumin data sets being required for the repeated measure ANOVA analyses, making the study unsuitable to function as a longevity experiment).

### Statistical analyses

Rat urine albumin excretions were analysed and are presented as both 24 h. excretion rates (which are free from laboratory errors in creatinine measurements) and albumin/creatinine ratios (which are free from laboratory errors in 24 h. urine volume collection and timing). Overall longitudinal rat albumin excretion rates and ratios were compared across the groups using repeated measures ANOVAs using the 'group', i.e. control, recuperated or PLP, as the between-subjects factor and the albumin measurement as the within-subjects factor on natural logarithmically-transformed data. Mauchly's test was used to assess sphericity and non-sphericity was corrected for using the Greenhouse-Geisser correction [11]. Post-hoc comparisons between the groups were performed using the Bonferroni correction. Where the analysis was cross-sectional rather than longitudinal due to only single measurements being taken, such as for kidney weight and creatinine clearance data, results were analysed using the Kruskal Wallis analysis of variance by ranks. Where there were only two groups to compare, such as for the body weights of the rats at 2 days of age, the Mann Whitney U test was used. P < 0.05 was considered statistically significant throughout. Due to much of the data not being normally distributed prior to transformation, data are shown as geometric mean (95 % confidence interval) unless otherwise stated. Statistical analyses were performed using SPSS for Windows (version 10.1.3; SPSS Inc., Chicago, U.S.A.).

## Results

### Body and kidney weights

**Figure 1:**
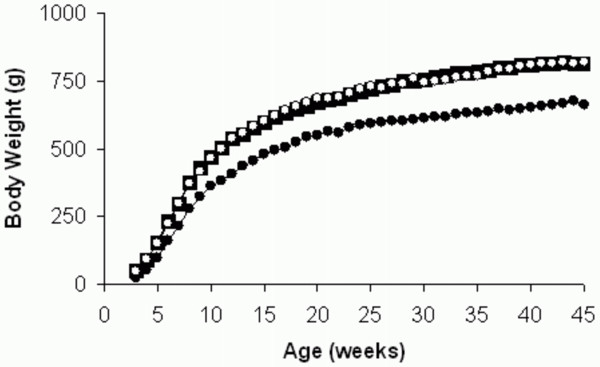
**Weights of rats whose mothers had been fed diets differing in their protein contents**. There were three groups of male rats: those whose mothers were fed a diet containing 20% protein throughout pregnancy and lactation ('control'; n = 24 animals from 6 different litters; relatively large solid squares), those that were born to dams fed the 8% protein diet but were subsequently cross-fostered by dams fed the 20% protein diet ('recuperated'; n = 16 animals from 5 different litters; open circles) and those that were born to control dams but were subsequently cross-fostered by dams fed a diet containing 8% protein ('post-natal low protein'; n = 20 animals from 5 different litters; solid circles). Data are medians.

Pups born to protein restricted mothers were significantly lighter at two days of age than those that were normally nourished (6.4 (6.0~7.3)g (n = 16 animals from 5 different litters) v. 7.0 (6.5~7.6)g (n = 44 animals from 11 different litters); p < 0.02). The weights of some of the male rats were recuperated using cross fostering techniques with dams fed the control diet. They exhibited rapid catch-up growth prior to weaning so that at 21 days of age their body weights were indistinguishable from those of the controls (50.7 (48.4~57.1)g (n = 16 animals from 5 different litters) v. 52.2 (47.3~56.0)g (n = 24 animals from 6 different litters), p = 0.8). After weaning their body weights continued to be indistinguishable from those of the controls throughout (Fig. 1). In contrast the PLP pups exhibited a slowing down of growth, so that by 21 days of age they were significantly lighter than the controls (24.8 (21.1~25.6)g (n = 20 animals from 5 different litters); p < 0.001). After weaning they continued to gain weight but at a lower trajectory than the other two groups (Fig. 1).

The first animal to die (at 10 months of age) came from the recuperated group. Kidney weights at 10 months of age in the PLP group were significantly lower than in the other two groups: (controls 5.51 (5.00~6.78)g (n = 24 animals from 6 different litters); recuperated 5.35 (4.67~5.64)g (n = 15 animals from 5 different litters); PLP 4.04 (3.90~4.21)g (n = 20 animals from 5 different litters); p < 0.001). This was still evident when the weights were corrected for body weights: (controls 0.67 (0.63~0.77)%; recuperated 0.61 (0.55~0.69)%; PLP 0.59 (0.57~0.63)%; overall p = 0.002; in both experimental groups a lower percentage of total body weights were attributable to the kidneys: recuperated p < 0.05, PLP p < 0.001).

### Urine albumin excretions and albumin/creatinine ratios

**Figure 2:**
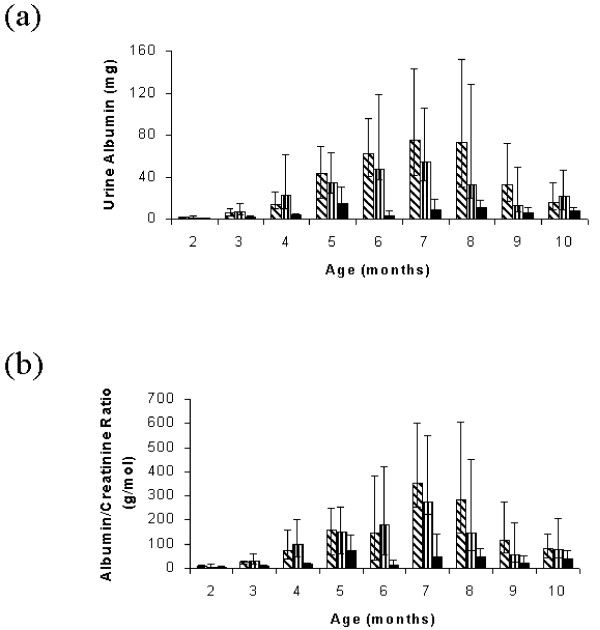
**Albuminuria levels in rats whose mothers had been fed diets differing in their protein contents**. There were three groups of male rats: those whose mothers were fed a diet containing 20% protein throughout pregnancy and lactation ('control'; n = 24 animals from 6 different litters; diagonal bars), those that were born to dams fed the 8% protein diet but were subsequently cross-fostered by dams fed the 20% protein diet ('recuperated'; n = 16 animals from 5 different litters; vertical bars) and those that were born to control dams but were subsequently cross-fostered by dams fed a diet containing 8% protein ('post-natal low protein'; n = 20 animals from 5 different litters; solid black bars). Fig. 2a shows daily albumin excretion rates and Fig. 2b shows albumin to creatinine ratios. Data are medians (interquartile ranges).

Fig. 2 shows the urine 24 h. albumin excretions and albumin to creatinine ratios in the three groups. Overall 24 h. urine albumin excretion rates (F = 97.82; Greenhouse-Geisser correction with 5.53 degrees of freedom, p < 0.001) and urine albumin to creatinine ratios (F = 56.39; Greenhouse-Geisser correction with 5.07 degrees of freedom, p < 0.001) were significantly different between the three groups. Estimated marginal geometric mean (95 % confidence interval) daily albumin excretions were: controls 19.5 (14.0~27.1) mg/day (24 animals from 6 different litters), recuperated 18.6 (12.4~27.8) (16 animals from 5 different litters), PLP 4.2 (2.9~6.0) (20 animals from 5 different litters). Control and recuperated animals therefore had significantly higher albuminuria excretion rates than PLP animals (both p < 0.001), but had albuminuria excretion rates that were indistinguishable from each other (p = 1.00). Estimated marginal geometric mean (95 % confidence interval) albumin to creatinine ratios were: controls 79.5 (57.2~110.6) g/mol (24 animals from 6 different litters), recuperated 71.0 (47.4~106.5) (16 animals from 5 different litters), PLP 21.2 (14.7~30.4) (20 animals from 5 different litters). Like with the 24 h. urine albumin excretions, control and recuperated animals had significantly higher urine albumin to creatinine ratios than PLP animals (both p < 0.001), but had ratios that were indistinguishable from each other (p = 1.00).

From three months of age there was a significant difference in urine albumin excretions (p < 0.001) and albumin to creatinine ratios (p < 0.001) between the groups, with the PLP rats having lower albumin excretions (p < 0.001) and albumin to creatinine ratios (p < 0.001) than control and recuperated animals (which themselves were indistinguishable p = 0.9 and 1.0, respectively). This trend continued until 10 months of age by which time there were no differences in either urine albumin excretions (p = 0.1) or albumin to creatinine ratios (p = 0.3). However, at this age the creatinine clearances of the PLP animals were significantly lower (p = 0.001) than those of the recuperated group (control group: 1.85 (1.60~2.23)ml/min.; recuperated group: 2.10 (2.02~2.19)ml/min.; PLP group: 1.65 (1.41~1.97)ml/min.; overall p = 0.006).

## Discussion

The present study has shown that in rats exposed to maternal isocaloric protein restriction during the first few weeks of life there were significant and long-lasting reductions in urinary albumin excretion that were detectable as early as three months of age. Previously such rats had been shown to have increased natural lifespans [1,2], especially in comparison to animals exposed to maternal protein restriction *in utero *who were subsequently cross-fostered by animals who were fed a diet containing a normal protein content. A role for the kidney in mediating the alterations in lifespans was suggested by the differences found in renal telomere lengths (unlike telomere lengths from the brains and livers), where relatively longer telomeres were found in kidneys from 13-month-old animals exposed to maternal protein restriction in the first few weeks of life [2]. The kidney appears to be particularly susceptible to effects associated with maternal protein restriction during pregnancy or lactation as in the present study we have confirmed the finding that maternal protein restriction during pregnancy leads to reductions in kidney weights that are greater than reductions in overall body weights [5] and others have demonstrated faster age-related deterioration in kidney function in offspring of pregnant rats fed a low protein diet that was supplemented with methionine [12]. Since albuminuria is able to cause renal damage [13-15], results from the present study would be entirely consistent with the suggestion that the increased lifespans and decreased renal telomere shortening observed in rats whose mothers were protein restricted whilst lactating might be directly attributable to the reduced albuminuria observed in such animals.

In the present study urinary albumin excretion in these rats exposed to maternal protein restriction whilst lactating appeared to be modest throughout life, whereas urinary albumin excretion in control rats and rats exposed *in utero *to maternal protein restriction, tended to increase with age up to around eight months and then drop to levels similar to those observed in the post-natally exposed rats. This pattern is different to what we have previously observed when using rats that had not been exposed to altered maternal diets and who were housed in individually ventilated cages [6]. These rats have considerably longer lifespans, however, in comparison to those used in the current study (unpublished observations), associated with being housed in a cleaner facility and therefore may well have experienced the drop in urine albumin excretion rates like that observed in the present study, just at an older age. Proteinuria (in particular albuminuria) has been traditionally used as a marker of glomerular leakage, but can also be an independent agent of renal damage [13-15]. As such, through a complex series of reactions [16], persistent heavy proteinuria may ultimately lead to renal inflammation and fibrosis. We have hypothesised that shortening of telomere lengths occurs through free radical production, with subsequent cellular senescence and finally apoptosis [6,17]. In fact the fall in albuminuria in the control and recuperated groups, at around nine months of age in the current study, may have been due to excessive fibrosis-related glomerular closure rather than a reduction in the leakiness of the remaining glomeruli.

The higher creatinine clearances at ten months of age in the recuperated group (who may possibly have reduced glomerular loads [18-20]) (relative to the rats exposed to post-natal maternal protein restriction) may reflect a hyperfiltration relative to that of the PLP rats, similar to what is seen early in the course of diabetic nephropathy [18,21] (which in itself is observed earlier in diabetics born with low birth weight who probably have fewer glomeruli [22]). In our rats, reducing maternal protein intake in early post-natal life appears to afford the kidneys long-lasting protection against future age-related disease. Reducing total calorie intake has long been known to reduce kidney pathology and increase lifespan in rats [23]. Our rats who were exposed to maternal protein restriction whilst suckling eat less than other rats after weaning [24], which may therefore explain their renal protection.

It has been hypothesised that the cycle of leaky glomeruli, proteinuria, cell damage including the formation of oxygen free radicals and a shortening of renal telomeres and further leakiness of glomeruli leads to apoptosis and fibrosis of nephrons [16]. Furthermore it was suggested that normally the removal of a few malfunctioning nephrons would be beneficial for survival since there is spare functional capacity of the kidney, which allows the well-functioning nephrons to survive and therefore overall kidney function to be maintained [16]. There would only be a problem if the spare functional capacity of the kidney were reduced, such as may be found in people (or animals) with low birth weight and a reduced complement of nephrons [19,20,25]. Such a mechanism is thought to explain the link between low birth weight and the earlier development of kidney disease in people with diabetes [22,26]. Whilst undoubtedly some of the mechanism thought to be involved in this programmed suicide of the nephron [16] has been gained from studies performed in rats, leading some to question its validity in humans [27], telomere shortening with age has previously been observed in human kidneys [28] enhancing its potential relevance. There are a number of other similarities between our rat model and humans which have exhibited catch-down post-natal growth, namely being both thinner [2,29] and more insulin sensitive [30,31]. A study of the effect of rapid post-natal weight gain against a background of poor fetal growth on long-term kidney function in humans would therefore appear to be important.

## Conclusion

This study has shown long-lasting diminished albumin excretion rates in adult rats exposed to maternal protein restriction whilst suckling as pups, using a rat model where the animals had previously been shown to have increased natural lifespans [1,2] and a slower rate of shortening of kidney DNA telomeres [2]. As albuminuria is an independent cause of nephron damage [13-15], the reduced albuminuria is likely to protect against age-related loss of renal function [16] in these rats and may contribute to their increased natural lifespans. Such findings are consistent with the "suicide of the nephron" hypothesis [16].

## Competing interests

The author(s) declare that they have no competing interests.

## Authors' contributions

CJP carried out the albumin ELISAs, performed the statistical analyses and drafted the manuscript. BJJ and LAJ performed the post-mortem organ collections and recorded the organ weights. CNH conceived the study, participated in its design and coordination and helped to draft the manuscript. SEO participated in the study design and helped draft the manuscript. All authors (except CNH) read and approved the final manuscript.

## Pre-publication history

The pre-publication history for this paper can be accessed here:



## References

[B1] Hales CN, Desai M, Ozanne SE, Crowther NJ (1996). Fishing in the stream of diabetes. From measuring insulin to the control of fetal organogenesis. Biochem Soc Trans.

[B2] Jennings BJ, Ozanne SE, Dorling MW, Hales CN (1999). Early growth determines longevity in male rats and may be related to telomere shortening in the kidney. FEBS Lett.

[B3] Ozanne SE, Hales CN (2004). Lifespan: catch-up growth and obesity in male mice. Nature.

[B4] Ozanne SE, Hales CN (2005). Poor fetal growth followed by rapid postnatal catch-up growth leads to premature death. Mech Ageing Dev.

[B5] Desai M, Crowther NJ, Lucas A, Hales CN (1996). Organ-selective growth in the offspring of protein-restricted mothers. Br J Nutr.

[B6] Tarry-Adkins JL, Ozanne SE, Norden A, Cherif H, Hales CN (2006). Lower antioxidant capacity and elevated p53 and p21 may be a link between gender disparity in renal telomere shortening, albuminuria, and longevity. Am J Physiol Renal Physiol.

[B7] Iwasaki K, Gleiser CA, Masoro EJ, McMahan CA, Seo EJ, Yu BP (1988). The influence of dietary protein source on longevity and age-related disease processes of Fischer rats. J Gerontol.

[B8] Peters LC, Kristal MB (1983). Suppression of infanticide in mother rats. J Comp Psychol.

[B9] Vienet R, Grognet JM, Ezan E, Lecaque D, Hamon G, Corman B (1994). Effect of chronic converting-enzyme inhibition on kidney function of senescent hypertensive rats. J Cardiovasc Pharmacol.

[B10] Kroll MH, Chesler R, Hagengruber C, Blank DW, Kestner J, Rawe M (1986). Automated determination of urinary creatinine without sample dilution: theory and practice. Clin Chem.

[B11] Greenhouse SW, Geisser S (1959). On methods in the analysis of profile data. Psychometrika.

[B12] Nwagwu MO, Cook A, Langley-Evans SC (2000). Evidence of progressive deterioration of renal function in rats exposed to maternal low-protein diet in utero. Brit J Nutr.

[B13] Walls J (2001). Relationship between proteinuria and progressive renal disease. Am J Kidney Dis.

[B14] Remuzzi G, Bertani T (1998). Pathophysiology of progressive nephropathies. New Eng J Med.

[B15] Thomas ME, Brunskill NJ, Harris KP, Bailey E, Pringle JH, Furness PN, Walls J (1999). Proteinuria induces tubular cell turnover: A potential mechanism for tubular atrophy. Kidney Int.

[B16] Hales CN (2001). Suicide of the nephron. Lancet.

[B17] Jennings BJ, Ozanne SE, Hales CN (2000). Nutrition, oxidative damage, telomere shortening, and cellular senescence: individual or connected agents of aging?. Mol Genet Metab.

[B18] Almeida JR, Mandarim-de-Lacerda CA (2005). Maternal gestational protein-calorie restriction decreases the number of glomeruli and causes glomerular hypertrophy in adult hypertensive rats. Am J Obstet Gynecol.

[B19] Zimanyi MA, Bertram JF, Black MJ (2000). Nephron number in the offspring of rats fed a low protein diet during pregnancy. Image Anal Stereology.

[B20] Sahajpal V, Ashton N (2003). Renal function and angiotensin AT1 receptor expression in young rats following intrauterine exposure to a maternal low-protein diet. Clin Sci.

[B21] Mogensen CE (1999). Microalbuminuria, blood pressure and diabetic renal disease: origin and development of ideas. Diabetologia.

[B22] Rossing P, Tarnow L, Nielsen FS, Hansen BV, Brenner BM, Parving HH (1995). Low birth weight. A risk factor for development of diabetic nephropathy?. Diabetes.

[B23] Wyndham JR, Everitt AV, Everitt SF (1983). Effects of isolation and food restriction begun at 50 days on the development of age- associated renal disease in the male Wistar rat. Arch Gerontol Geriatr.

[B24] Ozanne SE, Jennings BJ, Hales CN, Barker DJP (2000). Metabolic alterations following early growth retardation. Fetal Origins Of Cardiovascular And Lung Disease.

[B25] Hughson M, Farris AB, Douglas-Denton R, Hoy WE, Bertram JF (2003). Glomerular number and size in autopsy kidneys: the relationship to birth weight. Kidney Int.

[B26] Brenner BM, Chertow GM (1994). Congenital oligonephropathy and the etiology of adult hypertension and progressive renal injury. Am J Kidney Dis.

[B27] Ravnskov U (2001). Suicide of the nephron is preventable. Lancet.

[B28] Melk A, Ramassar V, Helms LM, Moore R, Rayner D, Solez K, Halloran PF (2000). Telomere shortening in kidneys with age. J Am Soc Nephrol.

[B29] Ong KK, Ahmed ML, Emmett PM, Preece MA, Dunger DB (2000). Association between postnatal catch-up growth and obesity in childhood: prospective cohort study. BMJ.

[B30] Ong KK, Petry CJ, Emmett PM, Sandhu MS, Kiess W, Hales CN, Ness AR, Dunger DB, ALSPAC study team (2004). Insulin sensitivity and secretion in normal children related to size at birth, postnatal growth, and plasma insulin-like growth factor-I levels. Diabetologia.

[B31] Moura AS, Caldeira Filho JS, de Freitas Mathias PC, de Sa CC (1997). Insulin secretion impairment and insulin sensitivity improvement in adult rats undernourished during early lactation. Res Commun Mol Pathol Pharmacol.

